# Accelerating the digital transformation of community midwifery during the COVID-19 pandemic

**DOI:** 10.18332/ejm/142571

**Published:** 2021-10-07

**Authors:** Victoria G. Vivilaki, Catherine Chronaki, Stavroula Barbounaki, Elena Petelos

**Affiliations:** 1 Department of Midwifery, School of Health and Care Sciences, University of West Attica, Athens, Greece; 2 HL7 Europe, Brussels, Belgium; 3 European Federation for Medical Informatics, Le Montsur-Lausanne, Switzerland; 4 Clinic of Social and Family Medicine, Faculty of Medicine, University of Crete, Heraklion, Greece; 5 Health Services Research, CAPHRI Care and Public Health Research Institute, Faculty of Health, Medicine and Life Sciences, Maastricht University, Maastricht, The Netherlands

**Keywords:** COVID-19 pandemic, digitally literate, digital transformation, health care systems, value-based midwifery, prenatal and postnatal virtual visits

The digital transformation of health systems represents a concrete challenge and an urgent priority across the world. At the same time, women of childbearing potential belong to a digitally literate generation comprising frequent users of digital tools, internet services, and social media, although disparities are, of course, present, with various determinants affecting the ability to access and use said tools and services. The midwives caring for these women are also much more likely to have some degree of familiarity with such tools and services and awareness regarding [the emerging digital transformation] the emerging digital transformation. Although they may not be structurally supported or empowered and/or may lack formal training on how to optimize or modify their practice, community midwives may offer digital tools or services to the women, on an individual or group level.

The coronavirus (COVID-19) pandemic has led to a dramatic increase in the volume of prenatal and postnatal virtual visits^1^. Indeed, women and their families seek to obtain timely care from the safety of their homes. Many community midwives saw their online visit volume rapidly rise over a very short period of time and had to radically adapt their practice in a manner for which neither themselves nor the women they cared for may have been prepared, oftentimes, without any support or guidance available to them. Community midwives rapidly organized themselves, often innovating in a disruptive manner, to start utilizing digital tools in their communication, to transform their practice from physical to virtual spaces, to change routines, and to ensure women were safe in terms of COVID-19, ensuring quality services were available to them^2^. On many occasions, utilizing online platforms and videoconferencing software proved to be a manner of interaction and practice that could not adequately support the midwife–woman communication or indeed the trust relationship that has to be build and maintained. Therefore, such solutions can only be considered temporary measures and certainly not representing sustainable practice and system change. At the same time, women and their families have come to trust or even prefer virtual visits given these represent a safe option during the COVID-19 pandemic, a time of high uncertainty and multiple risks, nevertheless systematic research on how mother and child outcomes are affected from the delivery of such care lags behind.

## What can such changes really offer now and in the post-COVID-19 period?

Such tools and services could potentially add value by facilitating personalization, reducing, if not eliminating, administrative complexity and increasing women’s experiences during and after pregnancy. In particular, emerging standards like the International Patient Summary (IPS) (https://international-patient-summary.net/) can provide an excellent starting point for the sharing of information and knowledge before, during, and after visits, empowering women and supporting their autonomy and self-care too. The perinatal period is the most critical period of development; with evidence emerging from all quarters and across geographical regions of women being disproportionately affected by the many issues of the COVID-19 pandemic new tools can contribute towards supporting, but, also monitoring practice and the outcomes for women and children for this critical period of development. Thus, the potential of the digital transformation needs to be realized, whilst ensuring risk minimization and sound investment, with new technologies and services that are embeded in virtual spaces requiring both assessment and appraisal.

## What are the challenges to accelerating this digital transformation?

The comparative range of digital services currently developed for and available in midwifery across the world remains limited, whereas infrastructure challenges, low literacy levels, and cultural norms may hinder change, resulting in a low level of utilization of digital technologies. Less than one in ten households in the European Union has no access to the Internet, whereas affordability represents access and equity issues; not all social groups are equally able to harness the potential of digital technologies, with 74–87% and lower at subnational-level able to do so, with intrapopulation variations across the European Region^3^.

Midwives, and many other primary care professionals, have a high workload and additional effort to tackle digital workload under underdeveloped frames in terms of best practice, and in the absence guidelines and hands-on training. In some cases, lack of any prior knowledge of familiarity with such tools compromises their ability to personalize care and actively support women. Furthermore, organizational processes need to be redesigned by exploiting the latest digital technologies so that they satisfy women’s and midwives’ service requirements, seamlessly and effectively to levels that exceed their expectations^4^.

Continuing to exert effort whilst under stress and fatigue, particularly as most women are disproportionately affected during the COVID-19 pandemic at home and at work with additional emerging issues of work–life balance, can further tax midwives, threatening their physical and mental wellbeing. These additional occupational risks to which they are exposed, may, for example, cause burnout and impaired performance, which in turn compromises the safety and wellbeing of the women they care for, their families and our communities at large^5^. Therefore, there is an urgent need to focus on digital learning and to measure and improve upon digital literacy, with sound supportive mechanisms and interprofessional engagement to avoid digital burnout. That would mean crossing the digital divide along with the women, the families, and the communities they support, picking up new digital skills, like creating a personal virtual room, safe digital spaces for professional exchanges, creating infographics to tackle the infodemic which poses a real threat in terms of sound public health decision-making, and ensuring they are sufficiently informed and empowered to take good care of their health. Embracing digital learning can be the most active way to create a buffer to protect community midwives from all the adverse effects of overexposure to virtual environments and excessive technology-use stress. It is, therefore, critical to invest in digital midwifery and appropriate training and supportive mechanisms, as these would benefit them individually, but would also represent a sound investment for the women, their families, and our communities^6^.

## Where should we invest and on what should we focus our efforts?

Online midwifery care is bound to become routine and, undoubtedly, to a large extent, it is here to stay and grow after the COVID-19 pandemic. On the other hand, there is a commitment to integrate digital health technology, especially in community midwifery care (electronic health records, infographics, virtual waiting rooms, virtual prenatal and postnatal classes, virtual breastfeeding classes, virtual counselling for antenatal smoking cessation and so many other important public health interventions by community midwives). In this context, ensuring conformance to digital health standards like the European Electronic Health Record Exchange Format^7,8^ and accessibility guidelines, while systematically measuring and investing in digital literacy, are key to ensuring the development of high-level quality digital midwifery services. Taking into account cultural barriers and old norms can also result in adapting interventions so as to ensure the best possible uptake of new solutions, with appropriate emphasis given to the nature of midwifery, key parameters in terms of organizational settings, and the need to consider gender-sensitive care across the board when developing such tools. Furthermore, ensuring that midwives are equipped with the necessary digital skills, may require new professional certification credentials and systematic monitoring of outcomes, both for practitioners and for the women they care for. Last, but not least, interdisciplinary and interprofessional education, including task shifting, may further contribute to alleviate the burden while ensuring safe care for women, for newborns, and for the delivery of all services across the perinatal period.

## What should we always keep in mind and ultimately aim for?

‘Value-based midwifery’ should be the basis to ensure that women and their families are protected and cared for in the most women friendly and engaging digital setting. Redefining it on a wider perspective, encompassing the European value of solidarity and with due consideration to all its aspects, would allow transformation of the practice of midwifery, contributing to better outcomes for women and children, better health for the whole family, more satisfied and resilient midwifery workforce, and concrete contribution to public health and societal resilience^9^.

To meet women’s expectations, community midwives must advance to digital value-based healthcare^10^, stimulate women-based technologies, and most of all –as always– midwives should become the strategic guardian for the women in this digital transformation^11^. A need to invest in harnessing the potential for digital public health wellness and care, including promotion and prevention, is key to ensuring the continuity of essential services and effectively counterbalancing the effects of the COVID-19 pandemic, as well as to succeeding in digital transformation of community midwifery.

## Data Availability

Data sharing is not applicable to this article as no new data were created.
